# Effect of Curcumin on Growth Performance, Inflammation, Insulin level, and Lipid Metabolism in Weaned Piglets with IUGR

**DOI:** 10.3390/ani9121098

**Published:** 2019-12-09

**Authors:** Yu Niu, Jintian He, Yongwei Zhao, Mingming Shen, Lili Zhang, Xiang Zhong, Chao Wang, Tian Wang

**Affiliations:** College of Animal Science and Technology, Nanjing Agricultural University, Nanjing 210095, China; niuyu0227@126.com (Y.N.); 15150537273@163.com (J.H.); zhaoyongweinjau@163.com (Y.Z.); 2017105056@njau.edu.cn (M.S.); zhanglili@njau.edu.cn (L.Z.); zhongxiang@njau.edu.cn (X.Z.); wangchao121@njau.edu.cn (C.W.)

**Keywords:** IUGR, curcumin, piglets, inflammation, insulin resistance, lipid metabolism

## Abstract

**Simple Summary:**

Intrauterine growth retardation (IUGR) has adverse influences on the growth performance and body metabolism of animals. Curcumin, a naturally occurring phenolic compound, has been proven to improve the growth of pigs. However, the studies related to the role of curcumin in treating IUGR piglets are not clear. Therefore, the purpose of our study was to investigate the role of curcumin on the growth, secretion of serum cytokines and hepatic metabolism of IUGR piglets. We found that IUGR piglets are accompanied by impaired growth, inflammation, and insulin resistance, as well as increased hepatic lipid concentrations. Curcumin supplementation improved growth and reduced hepatic inflammatory levels, thereby attenuating insulin resistance and decreasing the hepatic lipid concentration of IUGR piglets.

**Abstract:**

The possible causes of intrauterine growth retardation (IUGR) might stem from placental insufficiency, maternal malnutrition, inflammation in utero, and other causes. IUGR has had an adverse influence on human health and animal production. Forty weaned piglets with normal birth weights (NBWs) or IUGR were randomly divided into four treatments groups: NBW, NC (NBW with curcumin supplementation), IUGR, and IC (IUGR with curcumin supplementation) from 26 to 50 d. Levels of cytokines, glucose, and lipid metabolism were evaluated. IUGR piglets showed slow growth during the experiment. Piglets with IUGR showed higher levels of serum pro-inflammatory cytokines, insulin resistance, and hepatic lipid accumulation. Curcumin supplementation reduced the production of serum pro-inflammatory cytokines, attenuated insulin resistance and hepatic triglyceride, and enhanced the hepatic glycogen concentrations and lipase activities of IUGR piglets. The hepatic mRNA expressions of the insulin-signaling pathway and lipogenic pathway were influenced by IUGR and were positively attenuated by diets supplemented with curcumin. In conclusion, IUGR caused slow growth, insulin resistance, and increased hepatic lipid levels. Diets supplemented with curcumin improved growth, attenuated insulin resistance, and reduced lipid levels in the liver by regulating the hepatic gene expressions of the related signaling pathway in IUGR piglets.

## 1. Introduction

Intrauterine growth retardation (IUGR) always refers to the low weight of newborns, which induces slow growth and the development of the body and organs [1]. IUGR has become a common diagnosis in obstetrics and a major problem in animal husbandry [1,2]. Barker et al. [3] revealed the close relationship between an IUGR fetus and later developments in adult life. IUGR at different stages of pregnancy can lead to phenotypes characterized by low birth weight with a subsequent failure of infant growth, including impaired immunity [4], insulin resistance [5], hepatic steatosis, and other metabolic syndromes [6]. These phenotypes are closely associated with particular patterns of metabolic abnormalities in adult life. In livestock, animals have been found to have IUGR induced by naturally occurring or environment factors; these animals include goats, pigs, sheeps and other animals. Notably, IUGR is prevalent in pigs (and other multifetal mammals), for whom IUGR is reported to have permanent and negative impacts on neonatal birth weight, postnatal growth, development, and functions of liver [7,8]. The liver is the main organ involved in the metabolism of dietary nutrients and is easily influenced by IUGR during pregnancy. Previous studies also demonstrated that IUGR suckling piglets more commonly suffered from abnormal lipid metabolism and insulin resistance [5].

Curcumin (C_21_H_20_O_6_), discovered by Lampe and Milobedeska in 1910, is a kind of phenolic compound extracted from turmeric and used in foodstuff, beverages, and medicine [9]. Dietary curcumin supplementation also leads to a variety of improvements in food health and animal production. Xun et al. [10] found that dietary curcumin supplementation had beneficial effects on improving the growth performance, intestinal mucosal barrier integrity, morphology, and immune status of weaned pigs. The positive role of curcumin in subacute stress response has been well reported in pig research [11]. In recent years, Joongsun Kim et al. [12] revealed that curcumin can also improve epithelial cell survival and recovery in the skin in a mini-pig model.

Pigs, common domestic animals with multiple pregnancies, have been widely researched both for human health regulation and in animal nutrition. IUGR piglets exhibited the most severe naturally occurring conditions due to nutrition deficiencies during gestation [13]. However, studies that describe the treatment of curcumin on IUGR piglets are currently very limited. Therefore, our study chose weaned piglets with IUGR for our research model to investigate whether IUGR could impair the growth and secretion of serum cytokines, induce injury, and impair the metabolism of the liver, and to explore the effects of a diet supplemented with curcumin on the growth and metabolism of IUGR weaned piglets.

## 2. Materials and Methods

### 2.1. Curcumin Preparation

The curcumin supplements in the current study were offered by a commercial company for free (Kehu Bio-technology Research Center; Guangzhou, China). The content of curcumin was 98%, as determined by a high performance liquid chromatography (HPLC) analysis.

### 2.2. Animal Experiment Design

Forty newborn piglets from 20 sows (Landrace × Yorkshire) were obtained from Lihua Animal Husbandry Co., Ltd. (Changzhou, China). The diets [14] and management of the sows were similar during the gestation and suckling period. One piglet with a birth weight of 1.52 ± 0.03 kg (within one SD of the mean birth weight) and one piglet with a birth weight of 0.81 ± 0.03 kg (2 SD below the mean birth weight) in each litter were chosen as the normal birth weight (NBW) and IUGR piglets according to previous studies [15,16], respectively. Forty neonatal piglets (20 NBW and 20 IUGR, half male and half female) were selected after birth and labeled. During suckling, selected NBW and IUGR piglets were suckled by their mother sows until weaning.

All piglets were weaned on day 26. The twenty NBW piglets were averagely randomized into NBW and NC (curcumin diets) groups (half male and half female), and twenty IUGR piglets were averagely randomized into IUGR and IC (curcumin diets) groups (half male and half female). In each group, the piglets were assigned to 5 boxes (2 m × 2 m × 1.5 m; 2 animals in each box, one male and one female). The weaned piglets of the NBW and IUGR groups were fed with a control diet, and the weaned piglets of the NC and IC groups were fed with a curcumin diet (400 mg curcumin per kg control diet) from day 26 to day 50. Curcumin was added to the feed before it was made into pellets. The diets were supplemented with 400 mg/kg curcumin, according to the previous study [10], which demonstrated that dietary supplementation of 400 mg/kg curcumin was more effective in improving the health status of weaned pigs. The feeding schedule and temperature control were consistent with those of regular farm regulations. All the piglets were allowed food and water ad libitum during the experiment. The feed intake of the piglet in each box was recorded daily to calculate the average daily feed intake from 26 to 50 d of age. The body weights of the piglets were recorded at days 0, 7, 14, 21, and 26 to observe the growth of the piglets during suckling. At 50 d of age, the piglets were weighed, and the average daily gain (ADG), average daily feed intake (ADFI), and feed conversion ratio (FCR) were calculated similarly to previous studies [10]. The compositions of the diets are presented in Table 1.

### 2.3. Sample Collection

At 50 d of age, blood samples were collected after 12 h of fasting by jugular venipuncture before slaughter. Then, serum was obtained after centrifuging at 3000× *g* for 15 min at 4 °C and stored at 20 °C for further analysis. A total of 32 pigs with nearly similar body weights within the groups (8 pigs/group, half male and half female) were chosen and killed by jugular bloodletting after 12 h of fasting. Their organs were separated and weighed immediately to calculate the relative organ weights. The relative weight of the organ was equal to the organ weight (g) compared to the body weight (kg). Fresh liver samples were immediately collected and then stored at −80 °C for further analysis.

### 2.4. Serum Tumor Necrosis α, Interleukin 1β, and Interleukin 6

The concentration of serum tumor necrosis α (TNF-α), interleukin 1β (IL-1β), and interleukin 6 (IL-6) were measured by a commercial enzyme linked immunosorbent assay kits (Shanghai Yili biotechnology co., Ltd., Shanghai, China). The inter- and intra-assay coefficients of variation for each analyte are as follows: TNF-α (2.6%, 2.3%), IL-1β (1.5%, 1.3%), and IL-6 (3.2%, 2.9%).

### 2.5. Liver Aminotransferase Activities

The serum levels of aspartate aminotransferase (AST) and alanine aminotransferase (ALT) were determined according to a previous study (Selecta XL; Vital Scientific, Newton, MA, USA) [17].

### 2.6. Serum Insulin and Glucose

The levels of serum insulin and glucose were measured using commercial kits from Tianjin Nine Tripods Biomedical Engineering, Inc. (Tianjin, China). The inter- and intra-assay coefficients of variation for each analyte are as follows: glucose (3.5%, 3.3%) and insulin (4.3%, 4.1%). HOMA-IR = [fasting glucose (mmol/L) × fasting insulin (μU/mL)]/22.5; HOMA-IR, homeostasis model of assessment for the insulin resistance index.

### 2.7. Serum and Liver Biochemistry Parameters

The liver samples, stored at −80 °C, were homogenized according to the instructions of the manufacturer. Concentrations of total cholesterol (TC, A111-1) and triglyceride (TG, A110-1) in the serum and liver were analyzed using commercial kits (Nanjing jiancheng bioengineering institute, NJJC) [18,19]. Concentrations of high-density lipoprotein cholesterol (HDL-C, A112-1), low-density lipoprotein cholesterol (LDL-C, A113-1), glycogen (A043-1), pyruvate (A081), lactate (A019-2), non-esterified fatty acid (NEFA, A042), and the activities of hepatic lactic dehydrogenase (LDH, A020-1), pyruvate kinase (PK, A076-1), hepatic lipase (HL, A067), and lipoprotein lipase (LPL, A067) were determined using colorimetric kits (NJJC). The activity of total lipase (TL) was defined as the sum of the HL and LPL activities. The details of these testing kits manufacturer’s protocol (Nanjing Jiancheng Bioengineering Institute, Nanjing, Jiangsu Province, China) are clearly descripted in our supplemental files one by one [20].

### 2.8. Hepatic Gene Expression Assays

Hepatic total RNA was isolated using the TRIzol reagent (Invitrogen, Shanghai, China). The determination of RNA content, mRNA quantification, and real-time polymerase chain reaction (PCR; Applied Biosystems, Foster City, CA, USA) were performed similarly to previous reports [21]. The primer sequences for the target and housekeeping genes (*Irs1*, *Pik3c3*, *Akt2*, *Gsk3a*, *Gsk3b*, *Gys2*, *Fasn*, *Cd36*, *Fabp1*, *Lxrα*, *Ppara*, *Scd1*, *Srebp* and *Actb*) applied to real-time PCR are shown in Table 2. Briefly, a reaction system of 10 μL was composed of 0.2 μL of forward primers, 0.2 μL of reverse primers, 0.2 μL of ROX Reference Dye, 5 μL of SYBR Premix Ex Taq (TaKaRa Biotechnology Co. Ltd., Dalian, China), 3.4 μL of double-distilled water, and 1 μL of complementary DNA. The 2^−ΔΔCt^ method was used to calculate the relative levels of mRNA expression after normalization with housekeeping genes [22].

### 2.9. Statistical Analysis

The body weights of the piglets at 0, 7, 14, and 26 days of age and the average body weight gain of the piglets from 0 to 7, 7 to 14, 14 to 26, and 0 to 26 days of age were analyzed using unpaired independent *t*-tests. Other measurements were analyzed by a two-way analysis of variance. The classification variables were birth weight, curcumin diet, and the interaction between birth weight and the curcumin diet. A Tukey’s post hoc analysis was used to determine the differences between the four groups when a statistically significant birth weight × curcumin diet interaction was observed. SPSS 20.0 (SPSS, Inc., Chicago, IL, USA) was used for these analyses. A probability level of *p* < 0.05 was considered statistically significant, and *p* < 0.01 was considered highly significant. Data are presented as the mean ± standard deviation.

## 3. Results

### 3.1. Growth Performance

**Figure 1:**
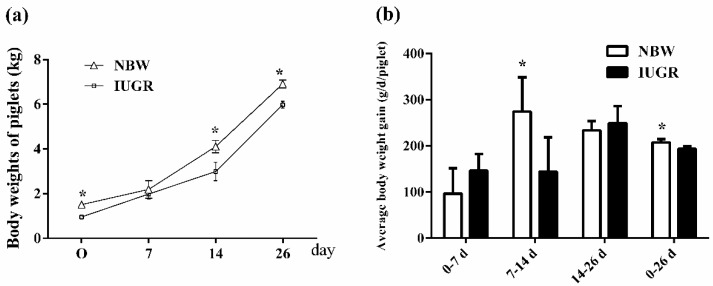
Analysis of body weight (**a**) and average body weight gain (**b**) in normal birth weight (NBW) and intrauterine growth retardation (IUGR) piglets during suckling from 0 to 26 days of age. Values are the means ± standard deviation; *n* = 20/group. Data were analyzed using unpaired, independent *t*-tests. * a significant difference was observed (*p* < 0.05). NBW, normal birth weight piglets; IUGR, intrauterine growth retardation piglets.

The body weights of the IUGR piglets were lower than those of the NBW piglets at 0 (*p* < 0.05), 7 (*p* > 0.05), 14 (*p* < 0.01) and 26 (*p* < 0.01) days of age, respectively (Figure 1a). The average body weight gain of the NBW piglets was lower than that of the IUGR piglets from 0 to 7 and 14 to 26 days of age (96.43 ± 54.79 vs. 146.55 ± 35.84, *p* > 0.05; 233.60 ± 19.88 vs. 248.82 ± 37.19, *p* > 0.05). The average body weight gain of the NBW piglets was higher than that of the IUGR piglets from 7 to 14 and 0 to 26 days of age (274.19 ± 74.15 vs. 144.29 ± 73.80, *p* = 0.01; 207.60 ± 6.66 vs. 193.14 ± 5.69, *p* < 0.01) (Figure 1b).

At 50 days of age, the IUGR weaned piglets showed a significantly lower (*p* < 0.05) final body weight (FBW), ADG, and ADFI compared to the NBW weaned piglets (Table 3). The FBW and ADFI of the IC group were increased (*p* < 0.05) compared to the IUGR group. The FCR of the NBW weaned piglets was significantly decreased (*p* < 0.05) after their diets were supplemented with curcumin.

### 3.2. Organ Index

The liver, kidney, and pancreas weights and relative pancreas weight (PRW) were significantly decreased (*p* < 0.05) in the IUGR group compared to those in the NBW group (Table 4). In the IC group, the kidney weight, relative spleen weight (SRW), and relative kidney weight (KRW) were higher (*p* < 0.05) than those of the weaned piglets with IUGR. The NC group showed a significantly lower (*p* < 0.05) pancreas weight and relative liver weight (LRW) and a significantly higher (*p* < 0.05) KRW compared to the NBW group.

### 3.3. Levels of Serum TNF-α, IL-1β and IL-6

The levels of serum TNF-α, IL-1β, and IL-6 in the IUGR group were significantly higher (*p* < 0.05) than those of the NBW group (Table 5). Diets supplemented with curcumin reduced the levels of serum TNF-α (*p* = 0.01) and IL-1β (*p* < 0.05) both in NBW and IUGR weaned piglets.

### 3.4. Activities of Serum AST and ALT

**Figure 2:**
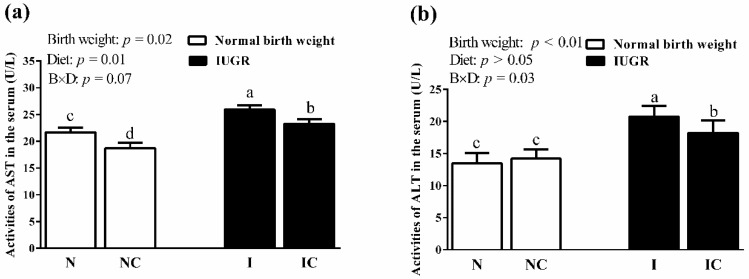
Influence of curcumin on the activities of serum AST (**a**) and ALT (**b**) among IUGR weaned piglets (50 d). Values are the means ± standard deviation; *n* = 8/group. B, birth weight of piglets; D, curcumin diets; B × D, the interaction between the birth weight and curcumin diets. ^abcd^ denotes where significant differences were observed (*p* < 0.05). N, piglets with normal birth weight and fed with control diets; NC, normal birth weight piglets fed with curcumin diets; I, piglets with intrauterine growth retardation and fed with control diets; IC, intrauterine growth retardation piglets fed with curcumin diets; AST, aspartate aminotransferase; ALT, alanine aminotransferase.

The activities of serum AST (Figure 2a) and ALT (Figure 2b) in the IUGR weaned piglets were significantly higher (*p* < 0.05) than those in the NBW weaned piglets. Diets supplemented with curcumin significantly decreased (*p* < 0.05) the activities of AST and ALT in the serum of IUGR weaned piglets. NBW piglets fed with curcumin diets also showed a significantly lower (*p* < 0.05) activity of serum AST than those of the NBW weaned piglets fed with control diets.

### 3.5. Serum Biochemistry Parameters

IUGR piglets exhibited significantly higher (*p* < 0.05) levels of serum insulin, HOMA-IR, and HDL-C, and significantly lower (*p* < 0.05) concentrations of serum NEFA compared to NBW piglets (Table 6). Diets supplemented with curcumin could significantly reduce (*p* < 0.05) the levels of insulin, glucose, HOMA-IR, and TC in the serum of the NC or IC groups compared to those of the NBW or IUGR groups. The concentration of HDL-C was reduced (*p* < 0.05), and the content of NEFA was increased (*p* < 0.05) more significantly in the serum of the IUGR weaned piglets fed curcumin diets than in the IUGR weaned piglets. Dietary curcumin supplementation enhanced (*p* < 0.05) the concentration of serum HDL-C and decreased (*p* < 0.05) the content of serum NEFA in NBW piglets.

### 3.6. Hepatic Biochemistry Parameters

Compared with those of the NBW piglets, the livers of IUGR weaned piglets exhibited significantly higher (*p* < 0.05) levels of TC, TG, NEFA, lactate and activities of pyruvate kinase, as well as significantly lower (*p* < 0.05) activities of LDH, LPL, HL, and TL and lower concentrations of glycogen (Table 7). Diets supplemented with curcumin significantly enhanced (*p* < 0.05) the activities of HL, TL, as well as the concentration of glycogen in the liver, and significantly decreased (*p* < 0.05) the activities of PK and the levels of TC, TG, NEFA, and pyruvate in the livers of the IC group. Diets supplemented with curcumin also enhanced (*p* < 0.05) the activities of LPL and TL and the levels of lactate and glycogen and decreased (*p* < 0.05) the activities of LDH, HL, and the levels of pyruvate, TG, and NEFA in the livers of the NBW group.

### 3.7. mRNA Expression

**Figure 3:**
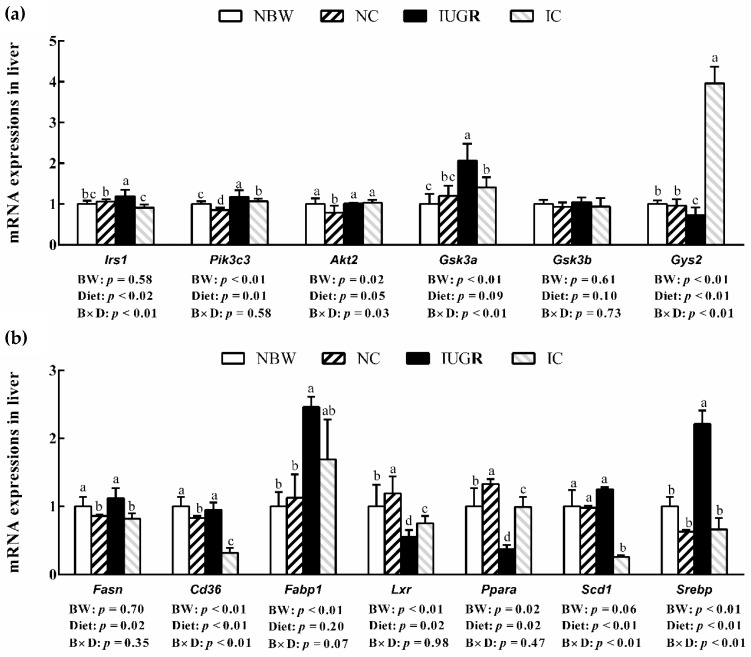
Influence of curcumin on the hepatic mRNA expressions of IUGR weaned piglets (**a**) insulin signal pathway, (**b**) lipid metabolism; 50 d). Values are the means ± standard deviation; *n* = 8/group. ^abcd^ denotes significant differences (*p* < 0.05). NBW, piglets with normal birth weights and fed with control diets; NC, NBW piglets fed with curcumin diets; IUGR, piglets with intrauterine growth retardation and fed with control diets; IC, IUGR piglets fed with curcumin diets; B, birth weight of piglets; D, curcumin diets; B × D, the interaction between the birth weight and curcumin diets. *Irs1*, insulin receptor substrate 1; *Pik3c3*, phosphatidylinositol 3-kinase catalytic subunit type 3; *Akt2*, serine/threonine kinase 2; *Gsk3a*, glycogen synthase kinase 3 alpha; *Gsk3b*, glycogen synthase kinase 3 beta; *Gys2*, glycogen synthase 2; *Fasn*, fatty acid synthase; *Cd36*, cluster of differentiation 36; *Fabp1*, liver fatty acid binding protein 1; *Lxrα*, liver × receptor; *Ppara*, peroxisome proliferator-activated receptor α; *Scd1*, stearoyl-CoA desaturase1; *Srebp*, sterol regulatory element binding proteins.

Compared to the NBW piglets, IUGR enhanced (*p* < 0.05) the hepatic *Irs1*, *Pik3c3*, *Gsk3a*, *Fabp1*, and *Srebp* mRNA expressions and reduced (*p* < 0.05) the hepatic *Gys2*, *Lxr* and *Ppara* mRNA expressions (Figure 3a,b). Diets supplemented with curcumin reduced (*p* < 0.05) hepatic *Irs1*, *Pik3c3*, *Gsk3a*, *Fasn*, *Cd36*, *Scd1*, and *Srebp* mRNA expressions and enhanced (*p* < 0.05) hepatic *Gys2*, *Lxr*, and *Ppara* mRNA expressions in the IC group. In the NC group, the hepatic *Pik3c3*, *Akt2*, *Fasn* and *Cd36* mRNA expressions were reduced (*p* < 0.05) and the hepatic *Lxr* and *Ppara* mRNA expressions were enhanced (*p* < 0.05) more than those of the NBW weaned piglets.

## 4. Discussion

IUGR always impairs the growth of the whole body and organ development in the early stage of life in piglets [23] and rats [24]. A previous study by He et al. [5] demonstrated that in suckling piglets, IUGR improves hepatic fatty acid synthase and leads to lipid accumulation in the liver. They also revealed that IUGR was easily accompanied by insulin resistance (IR) and abnormal lipid metabolism. However, studies related to the effects of curcumin on insulin resistance, hepatic glucose, and lipid metabolism in weaned piglets with IUGR are not well represented. Thus, IUGR weaned piglets were chosen as the animal model in our present study and we also studied the influences of diets supplemented with curcumin on the growth performance, insulin, hepatic glucose, and lipid metabolism of weaned piglets.

In our study, piglets with IUGR showed slow growth in the early period of life, as well as catch-up growth during the first week of age. However, IUGR piglets were still not able to achieve normal body weights, and this phenomenon lasted until 50 days of age in our study. Moreover, the BW and ADG were reduced in IUGR piglets after weaning. Our results suggest that IUGR might have a short period of catch-up growth after birth, as it was difficult for the pigs to reach normal levels. We also found that the weights or relative organ weights of IUGR weaned piglets were all decreased. Desai et al. [25] indicated that IUGR newborn animals always exhibit slow catch-up growth, impaired growth, and the development of organs with decreased weight [26]. These results are also similar to those of Dong et al. [23], who found that the weights of the body, spleen, and small intestines in IUGR suckling piglets were all reduced. Interestingly, diets supplemented with curcumin increased FBW, ADFI, and the relative weights of the spleen and kidney in IUGR weaned piglets. Previous studies demonstrated that dietary curcumin treatment could improve the negative growth performance caused by environmental factors [10,27]. In the present study, the effective role of curcumin on NBW weaned piglets was limited.

TNF-α and IL-1β are prototype pro-inflammatory cytokines involved in the pathogenesis of sepsis [28]. IL-6, a kind of pro-inflammatory cytokine, is also associated with continuous intrauterine hypoxia, which is a reason for the occurrence of IUGR [29]. In the current study, IUGR increased the levels of serum TNF-α, IL-1β, and IL-6 in weaned piglets, which might strongly suggest that inflammatory response existed in the piglets. We also found that IUGR piglets had a tendency to cause hepatic dysfunction and higher activities of serum AST and ALT. AST and ALT are usually located in the cytoplasm of liver. Once the liver is damaged, AST and ALT flow to the blood, and the high levels of these two activities in the serum have been widely accepted as biomarkers of hepatic damage. Our results are similar to those of the previous studies of Al-Azemi et al. [30] and Dacaj et al. [31] who demonstrated that increased pro-inflammatory cytokine dominance was observed in IUGR with placental insufficiency. Interestingly, high concentrations of serum pro-inflammatory cytokines, AST, and ALT in IUGR piglets were effectively attenuated after curcumin treatment. The positive role of curcumin on alleviating inflammation has been reported in a mouse model [32]. The results of the present study suggest that curcumin could reduce the inflammatory response and attenuate liver injury in IUGR piglets. Therefore, the beneficial effects of curcumin on the liver might be one of the main factors improving the growth performance of IUGR piglets.

The liver is an important metabolic organ in vivo, which regulates the stability of glucose and lipid metabolism between blood and the liver. It has been proven that IUGR can lead to changes of the liver involved in nutrient metabolism at the protein levels in fetal pigs [33]. Epidemiological studies also showed that IUGR is closely related to IR. A homeostasis model of assessment for the insulin resistance index (HOMA-IR) is considered a vital index of insulin resistance. In the present study, the levels of serum insulin and HOMA-IR were significantly increased in the IUGR piglets. This result suggests that IUGR might cause IR. These results are also similar to those of the previous study that showed IUGR suckling piglets to be associated with IR [5]. We also found that IUGR piglets increased the lactate content and decreased the glycogen concentration in the liver. Moreover, IUGR weaned piglets exhibited higher activities of pyruvate kinase and lactate dehydrogenase, while their pyruvate content remained unchanged. The decreased hepatic glycogen concentration in IUGR piglets might be related to the increased activity of pyruvate kinase. Pyruvate kinase is one of the main rate-limiting enzymes in glycolysis, which ultimately produces adenosine triphosphate and pyruvate in the metabolic process. These results indicate that IUGR accelerated glycolysis, leading to the decomposition of pyruvate and excessive lactate production. The increase of lactate in IUGR could produce a negative feedback regulating function for lactate dehydrogenase activity. We hypothesize that this might be the main reason for the dynamic balance of pyruvate in IUGR piglets. Li et al. [34] revealed that IUGR increased muscle glycolysis, leading to reduced glycogen synthesis and elevated lactate levels. After further investigation, IUGR up-regulated hepatic *Irs1*, *Pik3c3*, and *Gsk3a* mRNA expressions and down-regulated hepatic *Gys2* mRNA expression in weaned piglets in this experiment. In insulin signaling, insulin regulates glucose metabolism by combining with the insulin receptor, which activates *Irs1*, *Pik3c3* and *Akt2* and inhibits *Gsk3*, ultimately increasing the expression of *Gys2* and glycogen synthase [35]. Our results indicate that an increased insulin level activated hepatic insulin signaling in IUGR weaned piglets. However, hepatic *Gsk3a* mRNA expression was increased and *Gys2* mRNA expression was obviously decreased in the IUGR group, which then reduced hepatic glycogen synthesis. GSK3β, rather than GSK3α, is an important kinase that can regulate glycogen synthesis and promote glycogen deposition [36]. Morrison et al. [37] also reported that in order to make up for the deficiency of nutritional supply during the fetal period, IUGR neonates up-regulate the expressions of their insulin receptors and insulin signaling pathways after birth. In another study, the authors found that IUGR can permanently impair the insulin signaling pathway, increasing insulin levels and decreasing glycogen synthase in the liver [38]. Importantly, dietary curcumin supplementation significantly decreased insulin and glucose levels in IUGR weaned piglets, thereby reducing the risk of IR. Meanwhile, after curcumin treatment, the synthesis of hepatic glycogen was increased and the activity of pyruvate kinase was decreased in IUGR weaned piglets, thereby inhibiting glycolysis. We also found that a diet supplemented with curcumin down-regulated *Irs1*, *Pik3c3*, and *Gsk3a* mRNA expressions and up-regulated *Gys2* mRNA expression in the livers of IUGR weaned piglets. Previous studies have demonstrated that curcumin not only reduced insulin, glucose, and HOMA-IR levels [39] but also improved hepatic glycogen synthesis by promoting glucose absorption [40] and inhibiting glycogen synthase kinase activity [41]. These results demonstrate that curcumin plays a regulatory role in attenuating IR and maintaining glycogen homeostasis in the livers of IUGR weaned piglets. Curcumin was also beneficial in attenuating IR in NBW piglets.

In addition, this study shows that the concentration of serum HDL-C and concentrations of hepatic TC, TG, and NEFA were all enhanced in IUGR weaned piglets, and the content of serum NEFA and activities of hepatic HL and LPL were reduced in IUGR weaned piglets. The experimental findings suggest that TGs in the serum are transported to the liver in IUGR piglets. It has been reported that IR could accelerate NEFA transfer and lead to hepatic TG accumulation [42]. The decreased activities related to lipolysis in IUGR piglets are similar to the results of previous research on IUGR suckling piglets [5]. Musso et al. [43] reported that NEFA provides most of the lipid concentration for hepatic triglyceride synthesis in nonalcoholic fatty acid liver patients. They also observed that higher concentrations of hepatic NEFA and TG exist in IUGR. These results indicate that the accumulation of TG content and the increase of NEFA levels in the liver could be attributed to an imbalance of hepatic lipid metabolism [44]. Magee et al. [45] reported that the reason IUGR causes hepatic lipid accumulation might be related to the increase of liver fatty acid synthase and the inhibition of fatty acid oxidation. Our results show that IUGR up-regulated mRNA expressions for lipid synthesis (*Fasn*, *Fabp1* and *Srebp*) and down-regulated mRNA expressions for fatty acid oxidation (*Lxr* and *Ppara*). Other studies have also demonstrated that IUGR can up-regulate the mRNA expressions of a key transcription factor (*Srebp1c*) (and its downstream genes), and down-regulate the mRNA expressions of the fatty acid oxidation gene (*Ppara*), ultimately causing liver lipid metabolism disorder [46]. Lee et al. [47] found that IUGR, induced by maternal nutrition restriction, increased the protein expressions of SREBP1 and FASN. These results suggest that the abnormal lipid metabolism in the IUGR piglets was associated with related gene regulation. Previous studies have widely reported that curcumin treatment is beneficial in the regulation of abnormal lipid metabolism by decreasing the levels of serum and liver TC, TG, and free fatty acids [39], as well as down-regulating protein expressions of SREBP-1c and CD36 [39]. Interestingly, our results suggest that diets supplemented with curcumin decreased serum and liver TC and TG concentrations, inhibited NEFA exports to the liver from the serum, and increased the activities of HL, LPL, and TL in IUGR piglets. Curcumin also decreased the mRNA expressions of fatty acid transport (*Fabp1* and *Cd36*) and fatty acid synthesis (*Fasn*, *Scd1* and *Srebp*) and increased mRNA expressions of fatty acid oxidation (*Lxr* and *Ppara*) in IUGR piglets. Kang et al. [48] demonstrated that curcumin reduced TC and TG concentrations by decreasing the mRNA and protein expressions of SREBP-1 and FASN and increasing the mRNA and protein expressions of PPARα. According to the results of our present study, we confirm that curcumin can inhibit hepatic lipid synthesis and accelerate hepatic lipolysis and fatty acid oxidation in the livers of IUGR piglets. In the current study, curcumin also played a very important role in the regulation of inhibiting hepatic lipid accumulation in NBW piglets.

## 5. Conclusions

In summary, our present study demonstrates that IUGR impairs growth, causes inflammation and has a high risk of developing insulin resistance and abnormal lipid metabolism in the liver of weaned piglets. Curcumin supplementation obviously improved body weight and feed intake, reduced the levels of TNF-α and IL-1β, attenuated insulin resistance, and alleviated lipid accumulation by regulating the mRNA expressions of the insulin signaling pathway and lipid metabolism-related genes in the liver of IUGR piglets. Our findings suggest that dietary curcumin supplementation might be beneficial to improving anti-inflammation and glucose and lipid metabolism in IUGR humans and animal production.

## Figures and Tables

**Table 1:** Composition of the basal diets (as-fed basis).

Ingredients	Ratio (%)	Nutrient Composition	
Corn	57.70	Digestible energy (MJ/kg)	14.04
Soybean meal	12.50	Crude protein (%)	18.31
Expanded corn	8.00	Lysine (%)	1.31
Full-fat soybean	8.00	Methionine (%)	0.40
Fermented soybean meal	4.00	Methionine + Cystine (%)	0.70
Whey powder	3.00	Threonine (%)	0.80
Fish meal (crude protein 67%)	3.00	Calcium (%)	0.85
Dicalcium phosphate	1.80	Total phosphorus (%)	0.72
Limestone	0.50		
_L_-lysine (78%)	0.30		
_L_-threonine	0.10		
_DL_-methionine	0.08		
Wheat middling and reddog	0.02		
Premix *	1		
Total	100		

* The per kg complete diet included the following: vitamin A, 12,000 IU; vitamin D_3_, 3000 IU; α-tocopherol, 50 mg; vitamin K_3_, 4 mg; vitamin B_1_, 4 mg; vitamin B_2_, 10 mg; vitamin B_6_, 7 mg; vitamin B_12_, 0.05 mg; niacin, 30 mg; pantothenic acid, 15 mg; folic acid, 0.3 mg; biotin, 0.08 mg; choline chloride, 500 mg; Fe (FeSO_4_·H_2_O), 110 mg; Cu (CuSO_4_·5H_2_O), 7 mg; Zn (ZnO), 110 mg; I (KIO_3_), 0.3 mg; Mn (MnSO_4_·H_2_O), 5 mg; and Se (Na_2_SeO_3_), 0.3 mg.

**Table 2:** Primer sequences.

Genes	Accession No.	Primer, 5′-3′
*Irs1*	NM_001244489.1	GCCACGGGAGAATGGGTTTA GTCGCACACAGTTTCAGCAG
*Pik3c3*	XM_021093598.1	GCATGTTTCGCCAAGGGATG CTGCTTGTTCTGCCAGGAGT
*Akt2*	XM_011526619.1	CTGACCTGCTGTCCGCAAAT GACACGCTGTCACCTAGCTT
*Gsk3a*	XM_021093339.1	CAGTGCAAAGCAGTTGGTCC GGTGTAATCGGTGGCTCCAA
*Gsk3b*	NM_001128443	CGAGACACACCTGCACTCTT CCGGCATTAGTATCTGAGGCT
*Gys2*	NM_001195511.1	TGGGAATTCTGTGGGAAGCC TAGGTGCACTTGATGCAGGG
*Fasn*	NM_213839.1	TGATGCCCAAGTGACTGACC CAGCATGTTTCCGTTTGCCA
*Cd36*	NM_001044622.1	TAGGAATCCCACTGCCTCAC TGCTTCAAGTGCTGGGTCA
*Fabp1*	AY960623.1	GAGTAGCCTCATTGCCACCAT TGCACGATTTCCGATGTCCC
*Lxrα*	AB254405.1	CCCTCTCTCGCTCAGCTCC GGAGCCCTGGACATTACCAA
*Ppara*	NM_001044526.1	CTGGCCACATCCATCCAACA ATAACGGGCTTTCCAGGTCG
*Scd1*	NM_213781.1	TGCTGATCCCCACAATTCCC CTTTGACGGCTGGGTGTTTG
*Srebp*	AY338729.1	GCTACCGCTCCTCCATCAAT CTGCTTGAGCTTCTGGTTGC
*Actb*	XM_003124280.4	CAGTCGGTTGGATGGAGCAT AGGCAGGGACTTCCTGTAAC

*Irs1*, insulin receptor substrate 1; *Pik3c3*, phosphatidylinositol 3-kinase catalytic subunit type 3; *Akt2*, serine/threonine kinase 2; *Gsk3a*, glycogen synthase kinase 3 alpha; *Gsk3b*, glycogen synthase kinase 3 beta; *Gys2*, glycogen synthase 2; *Fasn*, fatty acid synthase; *Cd36*, cluster of differentiation 36; *Fabp1*, liver fatty acid binding protein 1; *Lxrα*, liver x receptor; *Ppara*, peroxisome proliferator-activated receptor α; *Scd1*, stearoyl-CoA desaturase1; *Srebp*, sterol regulatory element binding proteins; *Actb*, β-actin.

**Table 3:** Influence of curcumin on growth performance among IUGR weaned piglets (50 d).

Items	Experiment Groups				*p*-Value		
	NBW	NC	IUGR	IC	B	D	B × D
IBW (kg)	6.96 ± 0.13 ^a^	6.97 ± 0.34 ^a^	6.02 ± 0.17 ^b^	6.08 ± 0.05 ^b^	<0.01	0.60	0.68
FBW (kg)	13.01 ± 0.69 ^a^	12.55 ± 0.44 ^a^	10.48 ± 0.47 ^c^	11.38 ± 0.95 ^b^	<0.01	0.36	0.01
ADG (g/d/piglet)	251.85 ± 27.78 ^a^	257.91 ± 16.29 ^a^	183.59 ± 19.35 ^b^	204.04 ± 31.08 ^b^	<0.01	0.14	0.41
ADFI (g/d/piglet)	326.16 ± 13.78 ^a^	278.81 ± 20.74 ^a,b^	231.75 ± 17.84 ^b^	295.78 ± 17.95 ^a^	0.07	0.69	<0.01
FCR	1.33 ± 0.11 ^a^	1.12 ± 0.05 ^b^	1.36 ± 0.06 ^a^	1.39 ± 0.12 ^a^	<0.01	0.01	<0.01

Values are the means ± standard deviation; *n* = 5/group. Within a row, ^a,b,c^ denotes significant differences (*p* < 0.05). NBW, piglets with normal birth weight and fed with control diets; NC, NBW piglets fed with curcumin diets; IUGR, piglets with intrauterine growth retardation and fed with control diets; IC, IUGR piglets fed with curcumin diets; B, birth weight of piglets; D, curcumin diets; B × D, the interaction between the birth weight and curcumin diets. IBW, initial body weight; FBW, final body weight; ADG, average daily gain; ADFI, average daily feed intake; FCR, feed conversion ratio, ADG:ADFI.

**Table 4:** Influence of curcumin on the organ index among IUGR weaned piglets (50 d).

Items	Experiment Groups				*p*-Value		
	NBW	NC	IUGR	IC	B	D	B × D
Liver weight (g)	379.71 ± 22.42 ^a^	356.02 ± 29.56 ^a,b^	313.95 ± 23.26 ^c^	338.17 ± 26.40 ^b,c^	<0.01	0.98	0.01
Spleen weight (g)	26.44 ± 6.87	25.55 ± 5.23	21.28 ± 2.26	27.46 ± 7.02	0.42	0.20	0.09
Kidney weight (g)	58.81 ± 2.93 ^a^	58.85 ± 5.16 ^a^	49.24 ± 4.86 ^b^	56.24 ± 2.91 ^a^	<0.01	0.02	0.02
Pancreas weight (g)	31.28 ± 4.64 ^a^	24.96 ± 2.31 ^b^	22.74 ± 1.44 ^b^	22.99 ± 1.99 ^b^	<0.01	<0.01	<0.01
LRW (g/kg BW)	30.06 ± 2.11 ^a^	27.89 ± 1.44 ^b^	28.67 ± 1.65 ^a,b^	29.44 ± 2.42 ^a,b^	0.91	0.31	0.04
SRW (g/kg BW)	2.15 ± 0.18 ^b^	2.04 ± 0.15 ^b^	2.06 ± 0.26 ^b^	2.46 ± 0.14 ^a^	0.02	0.04	<0.01
KRW (g/kg BW)	4.77 ± 0.38 ^b^	5.01 ± 0.24 ^a^	4.78 ± 0.25 ^b^	5.39 ± 0.30 ^a^	0.08	<0.01	0.09
PRW (g/kg BW)	2.37 ± 0.37 ^a^	2.14 ± 0.22 ^a,b^	2.08 ± 0.20 ^b^	2.01 ± 0.15 ^b^	0.03	0.09	0.35

Values are the means ± standard deviation; *n* = 8/group. Within a row, ^a,b,c^ denotes significant differences (*p* < 0.05). NBW, piglets with normal birth weight and fed with control diets; NC, NBW piglets fed with curcumin diets; IUGR, piglets with intrauterine growth retardation and fed with control diets; IC, IUGR piglets fed with curcumin diets; B, birth weight of piglets; D, curcumin diets; B × D, the interaction between the birth weight and curcumin diets. LRW, the relative weight (RW) of liver; SRW, the RW of spleen; RKW, the RW of kidney; RPW, the RW of pancreas. BW, body weight.

**Table 5:** Influence of curcumin on the concentrations of serum pro-inflammatory cytokines in IUGR weaned piglets (50 d).

Items	Experiment Groups				*p*-Value		
	NBW	NC	IUGR	IC	B	D	B × D
TNF-α (pg/mL)	250.60 ± 24.10 ^b^	214.26 ± 51.03 ^c^	287.40 ± 29.56 ^a^	244.58 ± 19.13 ^b^	0.02	0.01	0.81
IL-1β (pg/mL)	747.52 ± 101.98 ^b^	674.78 ± 106.50 ^c^	856.67 ± 128.92 ^a^	743.36 ± 57.37 ^b^	0.05	0.04	0.63
IL-6 (pg/mL)	1162.44 ± 281.32 ^b^	899.94 ± 139.67 ^b^	1306.12 ± 400.78 ^a^	1208.26 ± 133.94 ^b^	0.05	0.11	0.45

Values are the means ± standard deviation; *n* = 8/group. Within a row, ^a,b,c^ denotes significant differences (*p* < 0.05). NBW, piglets with normal birth weights and fed with control diets; NC, NBW piglets fed with curcumin diets; IUGR, piglets with intrauterine growth retardation and fed with control diets; IC, IUGR piglets fed with curcumin diets; B, birth weight of piglets; D, curcumin diets; B × D, the interaction between the birth weight and curcumin diets. TNF-α, tumor necrosis factorα; IL-1β, interleukin 1β; IL-6, interleukin 6.

**Table 6:** Influence of curcumin on the levels of serum biochemistry parameters in IUGR weaned piglets (50 d).

Items	Experiment Groups				*p*-Value		
	NBW	NC	IUGR	IC	B	D	B × D
Insulin (mU/L)	19.44 ± 3.01 ^b^	16.77 ± 4.14 ^c^	22.71 ± 3.80 ^a^	19.64 ± 3.67 ^b^	0.03	0.04	0.88
Glucose (mmol/L)	5.14 ± 0.89 ^a^	4.53 ± 0.52 ^b^	5.75 ± 0.42 ^a^	4.27 ± 0.58 ^b^	0.44	<0.01	0.06
HOMA-IR	5.66 ± 1.06 ^b^	2.98 ± 0.51 ^d^	6.40 ± 1.06 ^a^	4.80 ± 1.55 ^c^	<0.01	<0.01	0.18
TC (mmol/L)	1.96 ± 0.36 ^a^	1.92 ± 0.08 ^b^	2.17 ± 0.04 ^a^	1.89 ± 0.09 ^b^	0.19	0.03	0.09
TG (mmol/L)	0.59 ± 0.13	0.55 ± 0.16	0.53 ± 0.17	0.50 ± 0.07	0.28	0.51	0.86
HDL-C (mmol/L)	1.23 ± 0.09 ^b^	1.55 ± 0.24 ^a^	1.59 ± 0.20 ^a^	1.32 ± 0.29 ^b^	0.42	0.75	<0.01
LDL-C (mmol/L)	0.86 ± 0.15 ^a^	0.71 ± 0.12 ^b^	0.67 ± 0.10 ^b^	0.85 ± 0.16 ^a^	0.63	0.71	<0.01
NEFA (μmol/L)	230.00 ± 21.46 ^a^	143.11 ± 34.15 ^c^	141.96 ± 18.10 ^c^	191.30 ± 21.03 ^b^	0.03	0.04	<0.01

Values are the means ± standard deviation; *n* = 8/group. Within a row, ^a,b,c,d^ denotes significant differences (*p* < 0.05). NBW, piglets with normal birth weight and fed with control diets; NC, NBW piglets fed with curcumin diets; IUGR, piglets with intrauterine growth retardation and fed with control diets; IC, IUGR piglets fed with curcumin diets; B, birth weight of piglets; D, curcumin diets; B × D, the interaction between the birth weight and curcumin diets. HOMA-IR = [fasting glucose (mmol/L) × fasting insulin (mU/L)]/22.5; TC, total cholesterol; TG, triglyceride; HDL-C, high density lipoprotein cholesterol; LDL-C, low density lipoprotein cholesterol; NEFA, non-esterified fatty acids.

**Table 7:** Influence of curcumin on the levels of hepatic biochemistry parameters in IUGR weaned piglets (50 d).

Items	Experiment Groups				*p*-Value		
	NBW	NC	IUGR	IC	B	D	B × D
Glycogen (mg/g tissue)	121.44 ± 20.58 ^c^	154.80 ± 22.24 ^b^	39.93 ± 18.72 ^d^	189.76 ± 37.56 ^a^	0.02	<0.01	<0.01
Pyruvate (μmol/mg prot)	0.04 ± 0.01 ^a^	0.03 ± 0.00 ^b^	0.04 ± 0.00 ^a^	0.03 ± 0.00 ^b^	0.31	<0.01	0.45
Lactate (mmol/g prot)	0.24 ± 0.04 ^b^	0.38 ± 0.11 ^a^	0.43 ± 0.07 ^a^	0.37 ± 0.10 ^a^	0.01	0.17	<0.01
PK (U/g prot)	17.17 ± 1.72 ^b^	18.63 ± 0.49 ^b^	22.27 ± 0.61 ^a^	18.59 ± 1.59 ^b^	<0.01	0.02	<0.01
LDH (U/g prot)	171.55 ± 23.53 ^a^	135.56 ± 13.76 ^b^	130.63 ± 8.68 ^b^	120.13 ± 14.11 ^b^	<0.01	<0.01	0.03
TC (μmol/g prot)	81.55 ± 19.51 ^b^	86.28 ± 13.92 ^b^	126.65 ± 22.83 ^a^	84.13 ± 12.52 ^b^	<0.01	0.01	<0.01
TG (μmol/g prot)	80.32 ± 6.44 ^b^	74.22 ± 5.34 ^c^	89.93 ± 9.12 ^a^	82.37 ± 8.93 ^b^	<0.01	0.02	0.79
NEFA (μmol/g prot)	36.06 ± 7.93 ^b^	24.85 ± 3.03 ^c^	50.19 ± 11.18 ^a^	22.09 ± 3.40 ^c^	0.03	<0.01	<0.01
LPL (U/mg prot)	0.16 ± 0.02 ^b^	0.23 ± 0.04 ^a^	0.08 ± 0.01 ^c^	0.10 ± 0.03 ^c^	<0.01	<0.01	0.03
HL (U/mg prot)	0.11 ± 0.02 ^a^	0.07 ± 0.01 ^b^	0.09 ± 0.01 ^b^	0.12 ± 0.03 ^a^	0.23	0.54	<0.01
TL (U/mg prot)	0.28 ± 0.03 ^b^	0.30 ± 0.04 ^a^	0.17 ± 0.01 ^d^	0.22 ± 0.04 ^c^	<0.01	<0.01	0.25

Values are the means × standard deviation; *n* = 8/group. Within a row, ^a,b,c,d^ denotes significant differences (*p* < 0.05). NBW, piglets with normal birth weights and fed with control diets; NC, NBW piglets fed with curcumin diets; IUGR, piglets with intrauterine growth retardation and fed with control diets; IC, IUGR piglets fed with curcumin diets; B, birth weight of piglets; D, curcumin diets; B × D, the interaction between the birth weight and curcumin diets. LDH, lactic dehydrogenase; PK, pyruvate kinase; TC, total cholesterol; TG, triglyceride; NEFA, non-esterified fatty acid.
